# A Plasmonic Approach to Study Protein Interaction Kinetics through the Dimerization of Functionalized Ag Nanoparticles

**DOI:** 10.1038/s41598-019-49583-2

**Published:** 2019-09-11

**Authors:** Pablo A. Mercadal, Ruben D. Motrich, Eduardo A. Coronado

**Affiliations:** 1 INFIQC-CONICET, Centro Láser de Ciencias Moleculares, Departamento de Fisicoquímica, Facultad de Ciencias Químicas, Universidad Nacional de Córdoba, Córdoba, Argentina; 2 CIBICI-CONICET, Departamento de Bioquímica Clínica, Facultad de Ciencias Químicas, Universidad Nacional de Córdoba, Córdoba, Argentina

**Keywords:** Nanoscale biophysics, Nanostructures

## Abstract

Understanding the kinetics of protein interactions plays a key role in biology with significant implications for the design of analytical methods for disease monitoring and diagnosis in medical care, research and industrial applications. Herein, we introduce a novel plasmonic approach to study the binding kinetics of protein-ligand interactions following the formation of silver nanoparticles (Ag NPs) dimers by UV-Vis spectroscopy that can be used as probes for antigen detection and quantification. To illustrate and test the method, the kinetics of the prototype biotin-streptavidin (Biot-STV) pair interaction was studied. Controlled aggregates (dimers) of STV functionalized Ag NPs were produced by adding stoichiometric quantities of gliadin-specific biotinylated antibodies (IgG-Biot). The dimerization kinetics was studied in a systematic way as a function of Ag NPs size and at different concentrations of IgG-Biot. The kinetics data have shown to be consistent with a complex reaction mechanism in which only the Ag NPs attached to the IgG-Biot located in a specific STV site are able to form dimers. These results help in elucidating a complex reaction mechanism involved in the dimerization kinetics of functionalized Ag NPs, which can serve as probes in surface plasmon resonance-based bioassays for the detection and quantification of different biomarkers or analytes of interest.

## Introduction

The binding affinities and kinetics of protein interactions play an important role to unravel biological processes^1–5^. Moreover, bioassays based on protein interactions are routinely used for diagnosis and clinical screening in medical care, research and also for biotechnology and industrial applications^6,7^. The methods to study protein interactions can be classified as substrate based^8,9^ or hydrodynamics free mixing methods^10,11^. In the first type of methods, the target molecules are attached to a substrate while in the second type the molecules are mixed in a suitable reactor. One of the standard methods of the first category is Surface Plasmon Resonance (SPR)^12,13^, frequently used for studying antigen-antibody interactions^14,15^, protein-DNA^16,17^ interactions and real-time analysis of bio-specific interactions without the use of labeled molecules^8,18,19^. On the same line, other methods recently developed include semiconductor nanowire field effect transistors, which have been applied as affinity biosensors able to measure protein-ligand interactions and their kinetics^20–24^. Typical approaches used to perform kinetic measurements in which both reactants are free to move are Stopped Flow^25–27^ and Hydrodynamic Focusing^28,29^. In the first one, large sample volumes are required making the method unsuitable for microassays. In order to overcome these difficulties, microfluidics techniques using hydrodynamic focusing were developed since they require small amounts of sample and a rapid mixing of the reactants^30,31^. The latter is achieved at high flow rates; therefore, one of the solutions must be used in excess for such a purpose^30^. Consequently, kinetics measurements that require varying the concentration of both reactants cannot be performed. Droplet based microfluidics has been recently introduced as an overcoming technique in which droplets can be generated into an immiscible carrier fluid^31–33^. Although the mixing process inside the droplet is not as fast as in Hydrodynamic Focusing, this kind of measurements has been demonstrated to be very competitive due to the low amounts of sample required and the precise control of the reaction and accurate monitoring of the time scale. Some notable examples in which this approach has been applied are the pioneering work for measuring the single turnover kinetics of ribonuclease A by monitoring the steady-state fluorescence arising from the cleavage of a fluorogenic substrate^34^ and the more recent study of the STV-Biot binding via fluorescence energy transfer (FRET)^32^.

In the present work, we introduce a novel technique in which the reporter event is the plasmonic coupling between two functionalized Ag NPs and the reactants are immobilized on Ag NP surface that are free to move in a colloidal dispersion. The spectral feature that probes the protein interaction is the formation of Ag NP dimers since each interacting moiety is located on the surface of each Ag NP monomer. The formation of Ag NP dimers leads to a relative weak plasmon coupling due to the relative large size of the proteins involved. This feature is evidenced by a depletion of the extinction intensity^35–37^, which allows to monitor the dimerization kinetics in a straightforward way and therefore to account for the binding kinetics. The difference between the present approach and previous methodologies is that molecules that form the dimeric structures are immobilized on an Ag NP surface, and the kinetics process occurs in the colloidal dispersion of the functionalized Ag NPs. As a prototype reaction, of upmost importance in many bioassays, we study the Biot-STV interaction by inducing the dimerization of STV functionalized Ag NPs through their conjugation with a biotinylated IgG antibody (IgG-Biot) specific for an antigen which detection/quantification may be of interest.

## Results and Discussion

**Figure 1:**
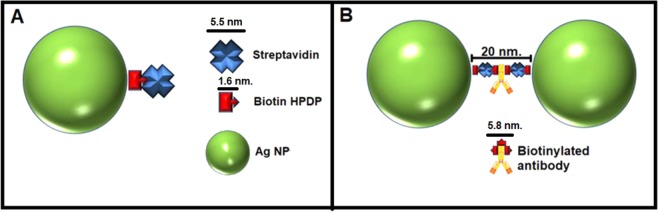
Schematic representation of the functionalization of Ag NPs and the formation of Ag NPs dimers. (**A**) Scheme of the Ag NPs functionalization with biotin-HPDP and streptavidin molecules. (**B**) Scheme of the formation of dimeric nanostructures in the presence of gliadin-specific biotinylated IgG with an interparticle distance of approximately 20 nm (estimated considering the geometry of the IgG antibody and the molecules attached to the Ag NPs).

The controlled dimeric formation is based on the functionalization of the Ag NPs surface with Biot (HPDP) and STV, leaving the surface of the Ag NP modified with the Biot-STV complex. In the latter, one of the STV sites is bound to biotin leaving the remaining 3 sites available for interacting with any biotinylated antibody. This strategy of bioconjugation using a 1:1:1 Ag NPs/Biot/STV molar relation modifies the Ag NP surface with approximately one molecule of STV/Biot complex per NP (Fig. 1A). The formation of dimers is induced by the addition of different concentrations of IgG-Biot, which acts as a molecular bridge between the STV of functionalized Ag NPs (Fig. 1B). Spectrally, the formation of dimers is evidenced by an intensity depletion of the extinction spectra. The main factors that explain why the formation of dimers is evidenced as a depletion of the extinction intensity are the lower values of the extinction cross section of a dimer compared with the extinction cross section corresponding to two monomers as well as the fact that the final concentration of NPs is smaller (note that in the limit where all the monomers form dimers the concentration of dimers is one half the concentration of monomers). In addition, the weak plasmonic coupling between the Ag NPs does not produce any significant change of the plasmonic peak position comparing the dimer and monomer spectra (Fig. S1).

Below, how the experimental decrease of the extinction intensity upon dimer formation can be used to determine the dimer concentration will be detailed. If the extinction depletion ${E}_{(D)}$, defined as the ratio between the extinction at any time *t*, ${E}_{t}\,$, and the initial extinction, ${E}_{0},$ that is ${E}_{(D)}=\frac{{E}_{t}}{{E}_{0}}$ is known, then the time dependent monomer and dimer concentrations for the global reaction 2M → D can be obtained from the dimer fraction, ${f}_{d}=\frac{[D]}{{[NPs]}_{t}}$, and the initial concentration of Ag NPs, ${[NPs]}_{i}$, as follows:

1 $$[M]={[NPs]}_{i}(\frac{1-{f}_{d}}{1+{f}_{d}})$$

2 $$[D]={[NPs]}_{i}(\frac{{f}_{d}}{1+{f}_{d}})$$

The extinction at a given time $t$ is given by the expression:

3 $${E}_{t}=[M]{\sigma }_{m}+[D]{\sigma }_{d}={[NPs]}_{i}(\frac{1-{f}_{d}}{1+{f}_{d}})+{[NPs]}_{i}(\frac{{f}_{d}}{1+{f}_{d}})$$

while the initial extinction is given by:

4 $${E}_{o}={[NPs]}_{i}{\sigma }_{m}$$

In the above equations, ${[NPs]}_{t}$ is the total nanoparticle concentration at time *t* (monomers plus dimers) while ${\sigma }_{m}$ and ${\sigma }_{d}$ are the monomer and dimer extinction cross sections, respectively.

The dimer fraction at time *t* can be obtained from the experimental extinction depletion and the calculated dimer and monomer extinction cross section, after replacing Eqs 3 and 4 in ${E}_{(D)}$:

5 $${f}_{d}=\frac{1-{E}_{D}}{{E}_{D}-\frac{{\sigma }_{d}}{{\sigma }_{m}}+1}$$

Finally, the concentration of dimers is obtained replacing Eqs (5) in (2):

6 $$[D]={[NPs]}_{i}(\frac{\frac{1-{E}_{D}}{{E}_{D}-\frac{{\sigma }_{d}}{{\sigma }_{m}}+1}}{1+\,\frac{1-{E}_{D}}{{E}_{D}-\frac{{\sigma }_{d}}{{\sigma }_{m}}+1}})={[NPs]}_{i}(\frac{1-{E}_{D}}{\frac{{\sigma }_{d}}{{\sigma }_{m}}+2})$$

As ${E}_{D}\,$ can be measured at each time *t* and the average ratio between the monomer and dimer extinction cross sections $\frac{{\sigma }_{d}}{{\sigma }_{m}}$ can be calculated using the Mie theory (for any NP size and at the wavelength of the maximum), it is therefore a straightforward task to determine the dimer concentration during the Ag NPs dimerization process.

**Figure 2:**
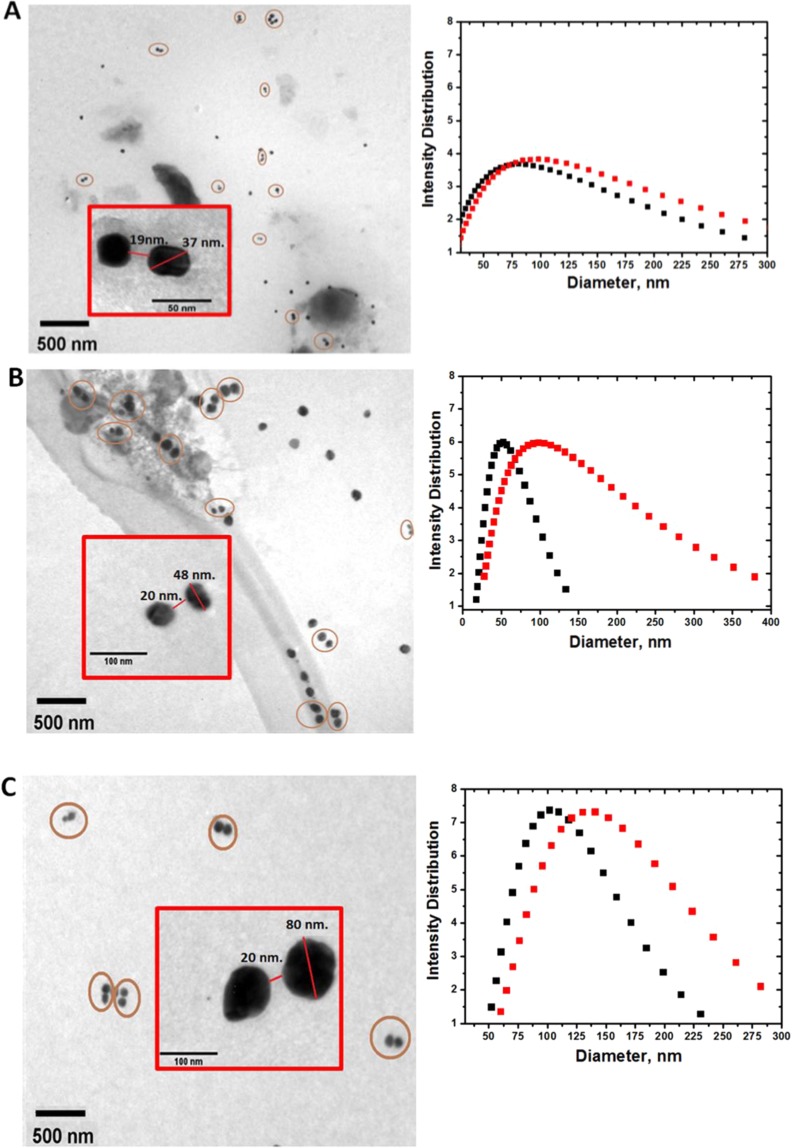
Transmission Electron Microscopy images and Dynamic Light Scattering response of the Ag nanostructures formed 30 minutes after adding different gliadin-specific biotinylated IgG concentrations. (**A**) TEM images of 38 nm diameter Ag NPs/0.75 ng/mL IgG-Biot and DLS analysis plot of the isolated Ag NPs. (**B**) TEM image of 46 nm diameter Ag NPs/0.5 ng/mL IgG-Biot and DLS analysis plot of the isolated Ag NPs. (**C**) TEM image of 76 nm Ag NPs/0.25 ng/mL IgG-Biot and DLS analysis plot of the isolated Ag NPs. Red squares show image magnifications of the Ag nanostructures formed 30 minutes after adding IgG-Biot.

In order to follow the dimerization kinetics, we have performed experiments using STV functionalized Ag NPs of three different average diameters (36 nm, 46 nm and 76 nm) and also varying the initial IgG-Biot concentration for each NP size. As shown in Fig. 2 (left panels), after 30 minutes of adding different quantities of IgG-Biot for three different Ag NPs sizes, transmission electron microscopy (TEM) analyses revealed that the Ag nanostructures formed are mostly dimers. Morphological features indicate that the interparticle distance is approximately 20 nm, in good agreement with the distance expected from the length of the Biot-STV-(IgG-Biot)-STV-Biot molecular bridge that connects the dimers. Moreover, Dynamic Light Scattering (DLS) measurements before and after adding the IgG-Biot to the Ag NPs colloidal dispersion qualitatively corroborated the formation of dimeric nanostructures (Fig. 2, right panels).

**Figure 3:**
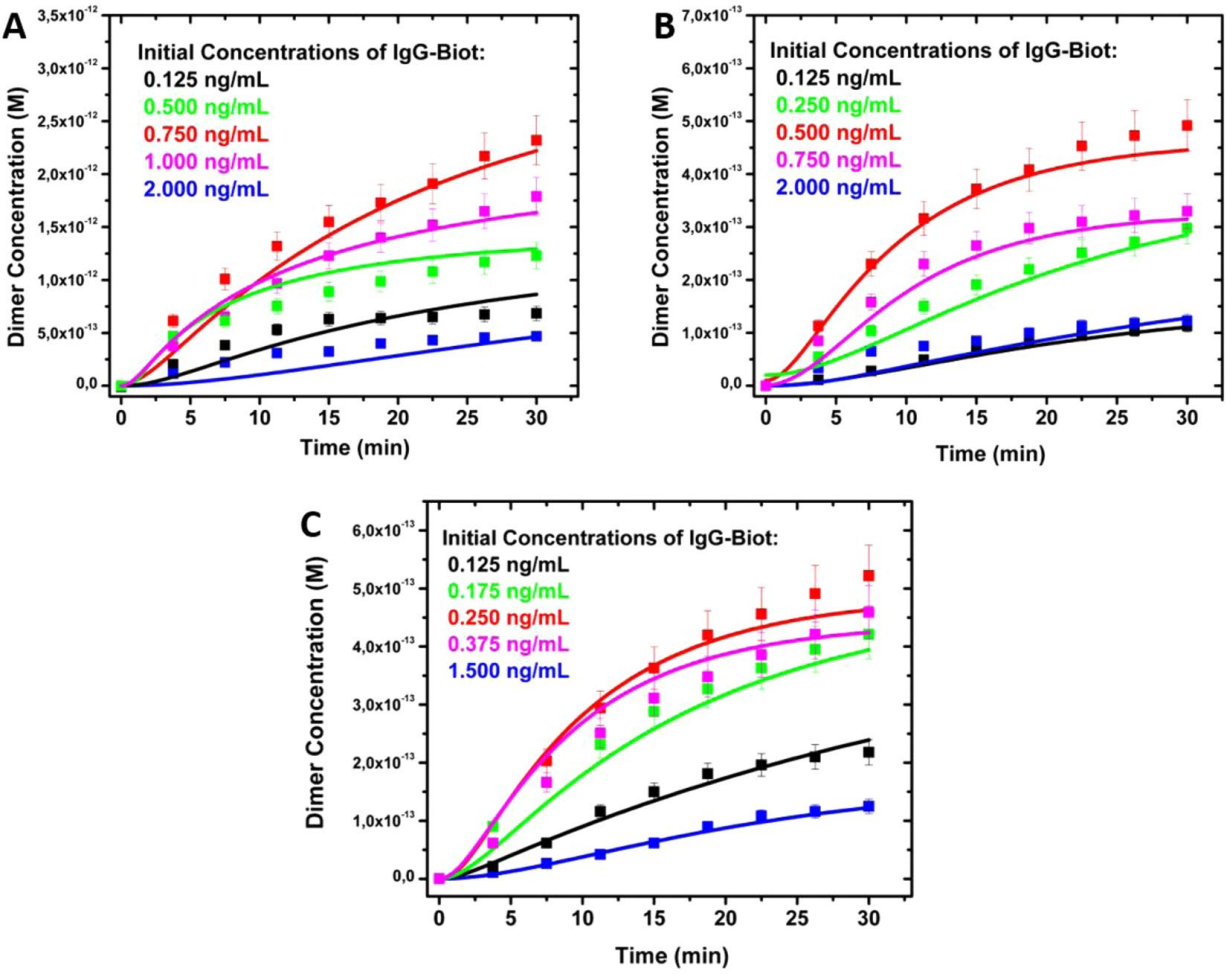
Experimental (squared symbols) and kinetics modelling (solid lines) of the evolution of the dimer concentration for different Ag NPs sizes and initial biotinylated IgG (IgG-Biot) concentrations. (**A**) 38 nm diameter Ag NPs, (**B**) 46 nm diameter Ag NPs, (**C**) 76 nm diameter Ag NPs. The initial IgG-Biot concentrations are indicated in the inset of each plot. The reaction mechanism and the kinetic rate constants are depicted in Table 1.

For each Ag NP size, the evolution of the extinction spectra was followed by UV-visible spectroscopy, from which the concentration of dimers at different times was determined using Eq. 6. Results of these experiments are shown in Fig. 3 for three different sizes of STV functionalized Ag NPs at five initial IgG-Biot concentrations. As shown, the dimer concentration increases with time until reaching an almost constant value at around 30 minutes (Fig. 3).

**Figure 4:**
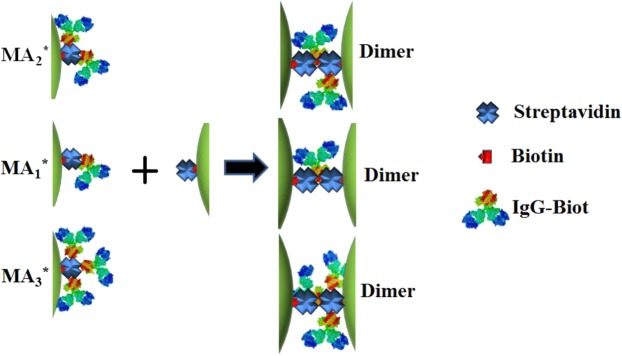
Representative scheme of the involved structures that lead to dimer formation in the proposed reaction mechanism for the interaction of gliadin-specific biotinylated IgG (IgG-Biot) functionalized Ag NPs with the reactive site of a STV functionalized Ag NPs.

**Figure 5:**
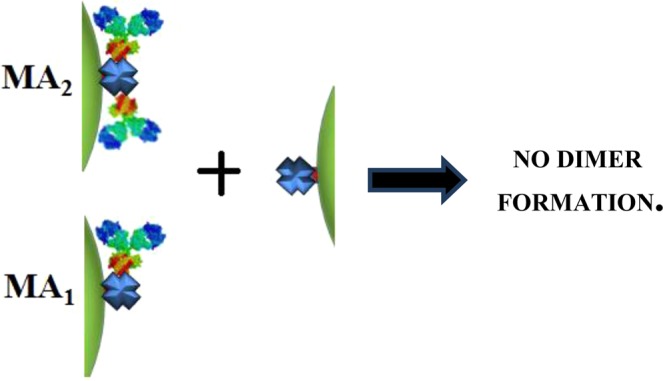
Representative scheme of the structures that are not allowed to form dimers in the proposed reaction mechanism for the interaction of gliadin-specific biotinylated IgG (IgG-Biot) functionalized Ag NPs with the STV functionalized Ag NPs.

The question that then arose was how the knowledge of the time dependent dimer concentration can be used to obtain information about the kinetics of the STV-Biot interaction. This issue could be solved taking into account that the concentration of dimers at any time *t* is the result of a complex reaction mechanism, which in principle should involve all the reaction steps depicted in Table 1. In this reaction mechanism, M denotes the STV-functionalized Ag NPs and A the IgG-Biot antibody. Three types of STV sites able to attach to the IgG-Biot have been distinguished. One denoted as “central” and the other two ones denoted as “side”. In a first stage, it is possible that IgG-Biot binds to either the central site or to any of the side sites, yielding the MA_1_* and MA_1_ nanostructures, respectively. A second antibody could also be attached to these two nanostructures giving rise to MA_2_* (if the central site has already been occupied by the first antibody or if the second antibody binds to the central site) or to MA_2_ (if both antibodies are located in the two “side” sites). These structures and the possible dimerization reactions are depicted in Figs 4 and 5. As it will be shown later, some of the reactions are not allowed (Fig. 5).

**Table 1:** Rate constants for each step of the site specific mechanism between STV functionalized Ag NPs (M) and biotinylated Ig-G antibodies (A).

Reaction	k(M^−1^ s^−1^) Ag NPs 38 nm	k(M^−1^ s^−1^) Ag NPs 46 nm	k(M^−1^ s^−1^) Ag NPs 76 nm
(1) M + A → MA_1_^*^	(6 ± 2) × 10^7^	(5 ± 3) × 10^7^	(6 ± 4) × 10^7^
(2) M + A → MA_1_	(4 ± 1) × 10^7^	(3 ± 2) × 10^7^	(5 ± 3) × 10^7^
(3) MA_1_^*^ + M → D	(5 ± 3) × 10^8^	(7 ± 4) × 10^8^	(4 ± 3) × 10^8^
(4) MA_1_ + M → D	not allowed	not allowed	not allowed
(5) MA_1_^*^ + A → MA_2_^*^	(4 ± 1) × 10^7^	(4 ± 3) × 10^7^	(3 ± 1) × 10^7^
(6) MA_1_ + A → MA_2_^*^	(6 ± 2) × 10^7^	(4 ± 3) × 10^7^	(5 ± 4) × 10^7^
(7) MA_1_ + A → MA_2_	(2 ± 1) × 10^7^	(3 ± 1) × 10^7^	(3 ± 2) × 10^7^
(8) MA_2_ + M → D	not allowed	not allowed	not allowed
(9) MA_2_^*^ + M → D	(4 ± 2) × 10^8^	(5 ± 1) × 10^8^	(4 ± 3) × 10^8^
(10)MA_2_^*^ + A → MA_3_^*^	(3 ± 2) × 10^7^	(1 ± 0.5) × 10^7^	(1 ± 0.4) × 10^7^
(11) MA_2_ + A → MA_3_^*^	(5 ± 1) × 10^7^	(1 ± 0.5) × 10^7^	(2 ± 1) × 10^7^
(12) MA_3_^*^ + M → D	(1 ± 0.6) × 10^7^	(1 ± 0.5) × 10^7^	(2 ± 1) × 10^7^

^*^Species with a biotinylated IgG (IgG-Biot) in the binding reactive site of STV.

The postulated (general) reaction mechanism was used as input to simulate the kinetics of dimer formation using the Kintecus software^38,39^. Note that the task consisted in fitting all the experimental curves of the evolution of the dimer (and monomer, data not shown) concentrations for a given NPs size and each initial antibody and NPs concentration with the same set of kinetics constants for each reaction step. Interestingly, if reactions (4) and (8) are allowed, it was impossible to fit the time evolution of D for all the experimental conditions (Fig. S2). This result is remarkable, since it shows the extreme sensibility of the time dependent dimer concentration to the specific reactions allowed in the general mechanism of dimer formation. Therefore, this kinetics modeling indicates that only certain configurations (marked with asterisks) in which the IgG-Biot is in the central site of STV moiety of the Ag NP are “active” or able to react to form dimers (Fig. 4). This site specificity seems quite reasonable since the other configurations shown in Fig. 5 are strongly not favored due to the steric hindrance between the location of the Biot moiety in the Fc region of the antibody in one NP and the relative large size of the second NP.

The set of allowed reaction rate constants for each step in this complex mechanism that are able to reproduce the experimental results (shown as solid lines in Fig. 3) are depicted in Table 1. All the reactions steps accounts essentially for the interaction between Biot and STV, and they are in the range of (70–0.4) × 10^7^ M^−1^s^−1^. These rate constants are in excellent agreement with previously reported data^20,32^. However, there are some important differences and similarities between the rate constants obtained in the present work that deserve to be highlighted and compared with recent works where the STV-Biot binding kinetics was measured in real time. Using a droplet microfluidics approach^32^, Srisa-Art *et al*. reported that the STV-Biot interaction is biphasic due to the multiple Biot binding sites on a STV molecule, with rate constants values of 4.5 × 10^7^ M^−1^s^−1^ and 3.6 × 10^6^ M^−1^s^−1^ associated to two different binding processes. The lowest value was explained in terms of the steric hindrance produced by the first Biot binding site since the biotin molecule was attached to a DNA strand. The kinetics data of the present work can be perfectly explained considering the same arguments. Comparing the rate constants of reactions (1), (5) and (10), the general trend is that the rate constant decreases as the successive sites of STV are being occupied by biotinylated antibodies, in an analogous way as previously observed by Srisa-Art *et al*.^32^. However and noteworthy, this effect seems to be less pronounced in the present work.

The steric hindrance effect on the STV-Biot rate constant can be appreciated more clearly when this interaction gives rise to Ag NP dimers. The rate constants for the dimerization process exhibit a significant decrease from values around 4–7 × 10^8^ to 1–2 × 10^7^ M^−1^s^−1^ comparing reactions (3) and (12) of Table 1. In reaction (3), the Ag NP is already bound to only one activated biotinylated antibody, while in reaction (12) the Ag NP is bound to three IgG-Biot antibodies, making the central active site less available and therefore leading to a smaller rate constant. An important arising fact is that the overall rate constants for reactions (3) and (9) are almost one order of magnitude greater than reaction (1) and (5) (which involves reactions between STV functionalized Ag NPs and IgG-Biot). These differences can be rationalized by comparing the rate constant obtained by microfluidics approaches^32^ with the affinity constant obtained using silicon nanowire biosensors^20^. In the first case, Biot and STV molecules are kept in a colloidal dispersion while in the second one Biot is bound to the surface of a silicon nanowire field effect transistor and STV is allowed to attach to this surface from an analyte solution. The highest values reported for the rate constants using the above mentioned methodologies^20,32^ are 4.5 × 10^7^ and 5.5 × 10^8^ M^−1^s^−1^, which also have an order of magnitude of difference, and similar to the results obtained in the present work for reactions (3) and (9). One possible explanation is that the probability of binding between STV and Biot not only depends on the steric hindrance but also on the freedom to move of both moieties (STV-Biot) in the system. If both entities are free to rotate, the number of events leading to a successful binding is lower with respect to the process in which one of the reactants is attached to a surface. To the best of our knowledge, this important effect (concerning molecular freedom) has not been addressed before in the kinetics of Biot-STV protein interaction.

Finally, another aspect that worth to be highlighted is the excellent agreement between the obtained experimental and simulated values for all the conditions shown in Fig. 3. That reflects the validity of the used kinetics modelling that further supports our conclusions.

The presented core conclusions as the role played by the steric hindrance in the incorporation of a second IgG-Biot as well as that the magnitude of the kinetic rate constant depends on the degree of mobility of the reactants provides a better understanding about protein interactions in nanostructures. The features highlighted above are of paramount importance for the development of new therapeutic protocols since they give information about the time-scale necessary to perform bioassays as well as the reaction mechanism involved. Furthermore, they could help for the design of robust, sensitive, and inexpensive methods for the detection of different biomarkers for disease monitoring and diagnosis in medical care, or other proteins of interest for research or industrial applications^6–12^. In this regard, some SPR based methods using metal NPs have been recently developed^7,10–17,33–35^. Among them, the intensity depletion immune-linked assay (IDILA) has been shown to be a promising alternative method for the inexpensive, rapid, accurate and ultrasensitive detection of disease biomarkers or food antigens in real samples^33–35^. This new sensing platform employs probes which consist in Biot-STV functionalized plasmonic Ag NPs linked to biotinylated antibodies specific to the protein/analyte of interest. Herein, we provide evidence that elucidates the molecular interactions involved in the functionalization of the Ag NPs with the specific antibody used as a probe in these bioassays.

One example where the present approach could be useful is for the development of methods to quantify antigens and antibodies. In this regard, it has been recently reported the synthesis of asymmetrically functionalized antibody-metal nanoparticles, with the aim to overcome the limitations of aggregation-based conjugates based immunoassays. These asymmetric conjugates assembled into dimers with the addition of antigen. The present methodology applied to this dimerization process should be able to elucidate the relevant kinetics information for the design of an immunoassay able to quantify antigens at very small concentrations, improving the performance of other aggregation-based immunoassays^40–44^.

## Concluding Remarks

In summary, in the present work we provided the kinetic parameters and elucidated a complex reaction mechanism that involves the ubiquitous Biot-STV interaction based on the dimerization kinetics of functionalized Ag NPs, which can serve as probes in SPR-based methods for the detection and quantification of different protein analytes. The relative large interparticle distance of the dimers produce an intensity depletion of the extinction spectra with respect to the isolated functionalized Ag NPs. This unique dimeric structure has an extinction cross section that peaks at almost the same wavelength of the monomer, with a value that is greater than the extinction cross section of two monomers, but less than twice. The above mentioned feature, together with the fact that the global nanostructure concentration (dimers plus monomers) decreases during the dimerization process, give rise to only a depletion of the peak extinction intensity.

From the extinction depletion as well as the initial NP concentration and the cross sections of the monomers and dimers, the evolution of $[D]$ can be easily obtained for any set of initial conditions. This information can be modelled with kinetics software by postulating a reaction mechanism, from which the reaction rate constants for the different steps involving protein interactions can be obtained. This novel strategy has been applied for studying the interactions of STV with a biotinylated antibody, both reactants immobilized on an Ag NP surface. It was found that only certain configurations of the IgG-Biot functionalized Ag NPs leads to the formations of dimers, i.e. only those where the antibody is located in a “central” or active site of STV. The rate constants obtained are in excellent agreement with previously reported data and lead to three important features concerning protein interactions:

- The role played by the steric hindrance in the incorporation of a second IgG-Biot to an already functionalized Ag NP with one antibody.
- The site specificity of the dimerization process.
- The fact that the magnitude of the kinetic rate constant depends on the degree of mobility of the reactants. This issue explains why the *k* values for NP dimerization are almost an order of magnitude grater (~10) than those corresponding to the reaction of a free IgG-Biot with a STV functionalized Ag NP. In the first process, both STV and the antibody are fixed to the NP surface and have less freedom to move, while in the second one only STV is fixed to the Ag NP.

These results are consistent with previous rate constants determined for STV-Biot interactions, but have not been highlighted before.

We anticipate that the present plasmonic approach based on the dimerization of functionalized Ag NPs could be a very versatile, simple and straightforward methodology to study protein interactions by optical means. Moreover, the present procedure can be adequately modified to study many relevant antigen-antibody interactions, which are of outmost importance to foster current understanding of many processes in cell biology, to improve drug discovery testing methods and affinity biodetection sensors for disease monitoring and diagnosis in medical care, research and industrial applications.

## Methods

### Reagents

The following reagents were used: AgNO_3_ (Blaker - Sigma Aldrich, St. Louis, MO, USA), sodium citrate (Anedra, Buenos Aires, Argentina), EZ-Link Biotin-HPDP (Pierce Biotechnology, Thermo Fisher, Rockford, IL, USA), streptavidin (Invitrogen, Carlsbad, CA, USA), biotinylated IgG antibody specific to wheat gliadin (LSBio Inc., Seattle, WA, USA), phosphate buffer solution, polysorbate 20 (Tween 20) (Sigma Aldrich), diluent solution [PBS containing 1% bovine serum albumin (BSA), Sigma Aldrich].

### Synthesis and functionalization of the Ag nanoparticles in colloidal dispersion

The synthesis of different Ag NPs size was performed by the Turkevich’s method^45^ adding 2.5, 1.75 or 1 mL of 0.01 M sodium citrate and 1 mL of 0.01 M Silver Nitrate solutions to 150 ml of boiling water under magnetic stirring. After 30 min, when a yellowish or yellow-white colour was observed the reaction was stopped. Silver NPs of average diameters of 36, 48, and 76 nm were obtained (Fig. S3). Using the Mie’s theory (to obtain the corresponding theoretical extinction cross sections (σ_Ext_)^46^ of diferent Ag NPs size) together with the Beer’s law, the following molar concentrations were obtained: 8.30 × 10^−12^ M, 3.58 × 10^−12^ M and 1.09 × 10^−12^ M for the 36, 46 and 76 nm Ag NPs average diameter colloidal dispersions, respectively.

For the surface modification of Ag NPs with streptavidin and Biotin-HPDP, Ag NPs of the three different sizes were incubated with STV/Biot in a 1: 1: 1 molar ratio (Ag NP-STV-Biot) for 1 hour at room temperature (Fig. S4).

### Extinction measurements

The optical characterization of Ag NPs was performed by UV-visible spectroscopy scanning in the 300–1100 nm wavelength range. The spectra obtained were measured using a Shimazdu UV-1700 PharmaSpec spectrophotometer (Shimadzu, Kyoto, Japan) with a 1 cm quartz cell at room temperature.

### Transmission electron microscopy

The morphological characterization of Ag NPs was performed by TEM using a JEM-JEOL 1120 EXII transmission electronic microscope (JEOL Ltd., Boston, MA, USA) under an accelerating voltage of 80 kV. Samples were prepared by adding one drop (∼30 μL) of the colloidal solution samples onto a holey carbon-formvar coated copper TEM grid (100 mesh).

### Dynamic light scattering

The characterization of the Ag NPs colloidal dispersion was also performed by DLS using a Delsa Nano 2.2 spectrophotometer (Beckman Coulter Inc., Brea, CA, USA) with a cell of 1 cm quartz at room temperature.

### Computational methods

The extinction spectra of isolated Ag NPs was simulated using the Mie’s theory and the dimeric nanostructures using the Mie’s theory generalized for multiple-particles (GMM)^35,46^. In all calculations performed, the NPs were excited by a plane wave with an incidence pointing vector (propagation direction) normal to the surface. All the extinction spectra of Ag NPs dimers were calculated using an interparticle spacing (gap distance) of 20 nm. The average extinction cross section was obtained by averaging the extinction cross sections that result from illuminating the dimer with polarizations around the x, y and z. ${\sigma }_{Ext}=\frac{1}{3}{\sigma }_{x}+\frac{1}{3}{\sigma }_{y}+\frac{1}{3}{\sigma }_{z}$. In all the simulations, the dielectric function tabulated by Palik^47^ for Ag was used. For the simulations of the extinction spectra of Ag NP monomers, the dielectric constant of the medium was of 1.77, corresponding to water, while for Ag NPs dimers a value of 1.87 was considered suitable due to the change produced by the addition of 1% BSA-PBS for the stabilization of the added antibody. The calculated extinction cross sections (${\sigma }_{ext}$) values for isolated Ag NPs with 38, 46 and 76 nm average diameters are 1.36 × 10^−10^ cm^2^, 1.87 × 10^−10^ cm^2^, and 4.40 × 10^−10^ cm^2^, respectively; while the ${\sigma }_{ext}$ for the respective dimers are 2.11 × 10^−10^ cm^2^, 2.84 × 10^−10^ cm^2^ and 7.14 × 10^−10^ cm^2^. All of these extinction cross sections were calculated at the wavelength corresponding to the maximum for each spectrum.

### Kinetic modelling

The kinetic modeling was performed using the Kintecus software^38^. The optimization of reaction mechanism was determined using, as input, the time evolution of both Ag NPs dimers as well as Ag NPs monomers for different IgG-Biot and Ag NPs initial concentrations. Different possible reaction mechanisms were explored, as detailed, and the rate equations were integrated using a time step of 10^−6^ s.

## Supplementary information

A Plasmonic Approach to Study Protein Interaction Kinetics through the Dimerization of Functionalized Ag Nanoparticles
