# Supplementary material for: A Plasmonic Approach to Study Protein Interaction Kinetics through the Dimerization of Functionalized Ag Nanoparticles
Source: Sci Rep. 2019 Sep 11;9:13122. doi: 10.1038/s41598-019-49583-2 (PMC6739483; doi:10.1038/s41598-019-49583-2)
Supplement: Supplementary file 1 — A Plasmonic Approach to Study Protein Interaction Kinetics through the Dimerization of Functionalized Ag Nanoparticles [file 41598_2019_49583_MOESM1_ESM.docx]

**SUPPLEMENTARY INFORMATION**

**A Plasmonic Approach to Study Protein Interaction Kinetics through the Dimerization of Functionalized Ag Nanoparticles**

*Pablo. A. Mercadal†, Ruben D. Motrich‡, and Eduardo A. Coronado*†.*

†INFIQC-CONICET, Centro Láser de Ciencias Moleculares, Departamento de Fisicoquímica, Facultad de Ciencias Químicas, Universidad Nacional de Córdoba, Córdoba, Argentina

‡CIBICI-CONICET, Departamento de Bioquímica Clínica, Facultad de Ciencias Químicas, Universidad Nacional de Córdoba, Córdoba, Argentina

Email: [coronado@fcq.unc.edu.ar](mailto:coronado@fcq.unc.edu.ar)


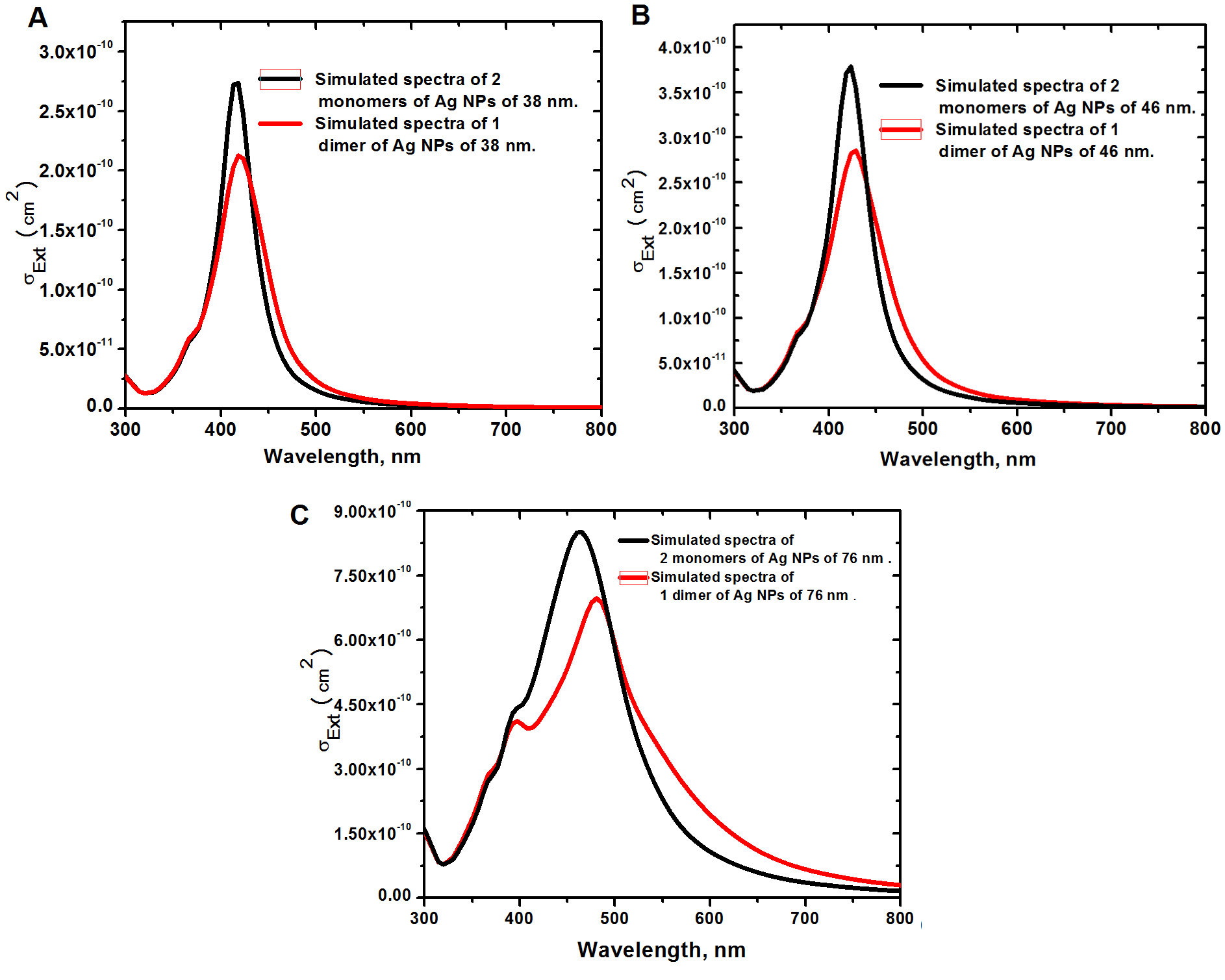


**Figure S1**. Theoretical modelling using the Mie’s theory and GMM theory of the extinction cross section of 2 monomers (black line) and the corresponding dimer averaged over x, y and z polarizations (red line) for (**A**) 38 nm average diameter Ag NPs, (**B**) 46 nm average diameter Ag NPs, and (**C**) 76 nm average diameter Ag NPs.





**Figure S2.** Representative experiment showing the total failure in fitting the experimental (squared symbols) and the kinetics (solid lines) modelling of the time evolution of dimer formation for 46 nm diameter STV functionalized Ag NPs at different initial biotinylated IgG (IgG-Biot) concentrations. In this example, it was considered that all the reactions between IgG-Biot functionalized Ag NPs were allowed to form dimers (reactions 3, 4, 8, 9 and 12 in the initial reaction mechanism proposed in Table 1).

**
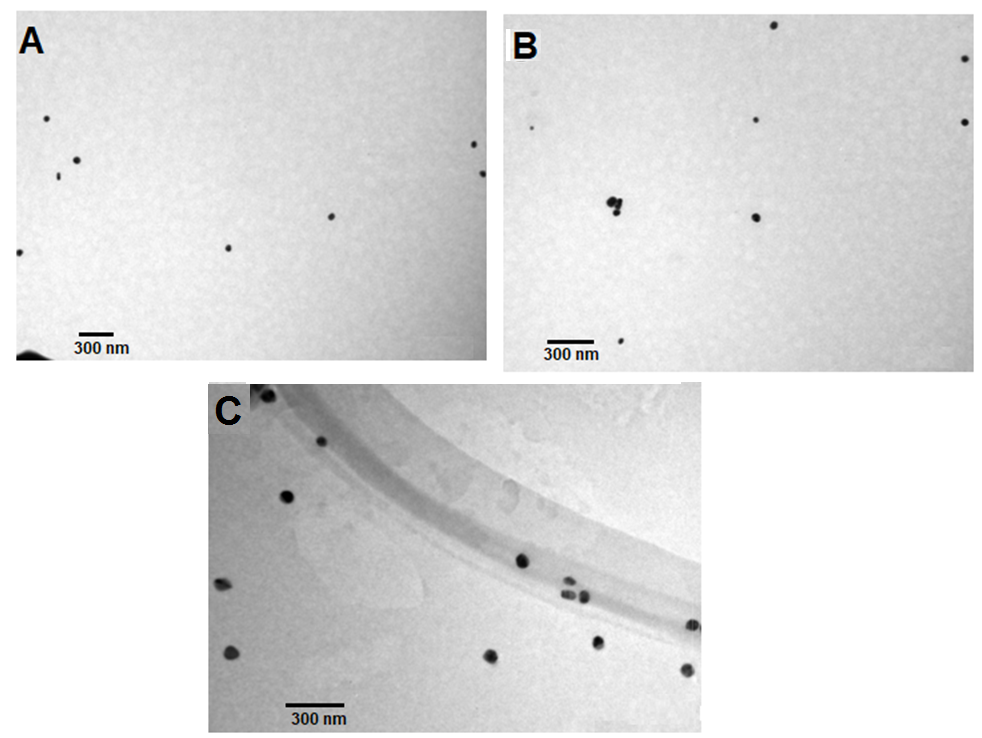
**

**Figure S3.** Representative Transmission Electron Microscopy (TEM) images of isolated Ag NPs sinthetized by the Turkevich´s method. (**A**) 38 nm diameter Ag NPs. (B) 46 nm diameter Ag NPs. (C) 76 nm of diameter Ag NPs.


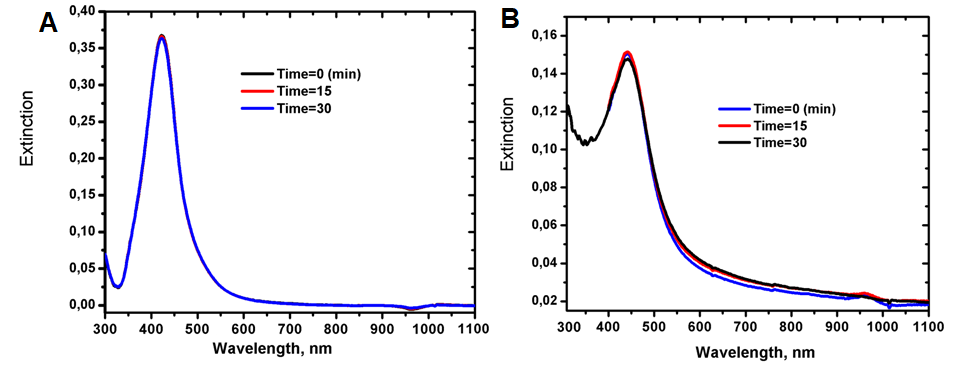


**Figure S4.** Stability of the colloidal dispersion of Ag NPs functionalized with STV-Biot with a 1/1/1 Ag NP-Biot-STV molar ratio. (**A**) 46 nm average diameter Ag NPs. (**B**) 58 nm average diameter Ag NPs.
